# Cretaceous chewing-louse eggs on enantiornithine birds

**DOI:** 10.1093/nsr/nwae479

**Published:** 2025-01-07

**Authors:** Chenyang Cai, Kevin P Johnson, Yanzhe Fu, Daniel R Gustafsson, Dany Azar, Yitong Su, Qiang Xuan, Michael S Engel, Diying Huang

**Affiliations:** State Key Laboratory of Palaeobiology and Stratigraphy, Nanjing Institute of Geology and Palaeontology and Center for Excellence in Life and Paleoenvironment, Chinese Academy of Sciences, China; Illinois Natural History Survey, Prairie Research Institute, University of Illinois, Urbana-Champaign, USA; State Key Laboratory of Palaeobiology and Stratigraphy, Nanjing Institute of Geology and Palaeontology and Center for Excellence in Life and Paleoenvironment, Chinese Academy of Sciences, China; Guangdong Key Laboratory of Animal Conservation and Resource Utilization, Guangdong Public Library of Wild Animal Conservation and Utilization, Institute of Zoology, Guangdong Academy of Sciences, China; State Key Laboratory of Palaeobiology and Stratigraphy, Nanjing Institute of Geology and Palaeontology and Center for Excellence in Life and Paleoenvironment, Chinese Academy of Sciences, China; State Key Laboratory of Palaeobiology and Stratigraphy, Nanjing Institute of Geology and Palaeontology and Center for Excellence in Life and Paleoenvironment, Chinese Academy of Sciences, China; State Key Laboratory of Palaeobiology and Stratigraphy, Nanjing Institute of Geology and Palaeontology and Center for Excellence in Life and Paleoenvironment, Chinese Academy of Sciences, China; Division of Invertebrate Zoology, American Museum of Natural History, USA; State Key Laboratory of Palaeobiology and Stratigraphy, Nanjing Institute of Geology and Palaeontology and Center for Excellence in Life and Paleoenvironment, Chinese Academy of Sciences, China

Ectoparasitism among insects has evolved innumerable times, exploiting hosts as varied as arachnids to vertebrates. The most infamous of ectoparasitic insects are those that live and feed on warm-blooded vertebrates. These parasites can negatively impact the fitness of their hosts by consuming blood, feathers or other external tissues. Few fossils of vertebrate ectoparasites are known [1], limiting direct insights into the historical presence and interactions of these parasites. While it has been suggested that diverse blood-feeding ectoparasites existed in the Jurassic and Cretaceous periods [1], definitive feather-feeding ectoparasitic insects and chelicerates from the Mesozoic are rare [1–3], and this is of particular interest given that many dinosaurs are known to have possessed feathers [4]. An enigmatic lineage of wingless insects (Mesophthiridae), preserved adjacent to partially damaged feathers, were initially interpreted as feather-feeding ectoparasites [5]. However, reinterpretation of their morphology and taphonomy indicates that these fossils are actually early instars (crawlers) of scale insects, a lineage that is obligately plant-feeding [6]. Among extant ectoparasites, lice (Phthiraptera) are the most diverse group of feather-feeding insects and have garnered considerable attention due to their ecological relationships and medical and veterinary significance. However, the fossil record of parasitic lice is scarce [7]. Wappler *et al.* [8] reported the first fossil chewing louse (Amblycera) from the Eocene Eckfeld maar (ca. 44 Ma) in Germany. This specimen contained preserved feather barbules within its foregut, serving as direct evidence of feather feeding. Recently, a new lineage of stem chewing lice was described based on two adults associated with semiplume feathers in mid-Cretaceous amber (ca. 99 Ma), pushing back the origin of ectoparasitic lice by over 55 million years [9]. Here we report the earliest fossil record for louse nits (eggs; [7,10]) tightly attached to enantiornithine (stem bird) feathers from the same deposit as the fossil adults [9], providing new evidence regarding the early origins of lice and their association with vertebrate hosts.

We examined dozens of amber-entombed feathers in our collection (ca. 40 000 fossiliferous Kachin amber pieces), uncovering a single specimen (Fig. S1a) preserved with two rows of eggs in a distinct arrangement, adhering tightly to two slender feather barbs (Fig. 1a). On one barb, six eggs, each ∼512 μm in length and 221 μm in width, were spaced at intervals of 526–748 μm, firmly attached to the rachis (Fig. 1b). Similarly, on the other barb, at least five eggs are arranged in a comparable pattern of attachment to the rachis (Fig. 1c). Notably, all isolated and downy barbs were found in close proximity within a single amber piece (Fig. S1b), indicating that they were derived from feathers of a single theropod individual. The eggs were enclosed in elongate chorions (Fig. 1d–g), oriented apically without a sheath cap (Fig. 1f and g). Based on the shape, size and arrangement of these fossil eggs, we suggest that they probably belonged to chewing lice, akin to modern-day bird lice. The unique morphology of the barbs and downy barbs (Fig. S2) bears a striking resemblance to those found on the wings of Enantiornithes [11], the most diverse stem birds in the Mesozoic, particularly those found in mid-Cretaceous Kachin amber [11,12]. Given the absence of other birds or definitive theropod dinosaurs in the amber deposit, we suggest that Cretaceous enantiornithine birds were parasitized by an early lineage of avian chewing lice (Fig. 1h).

**Figure 1.**
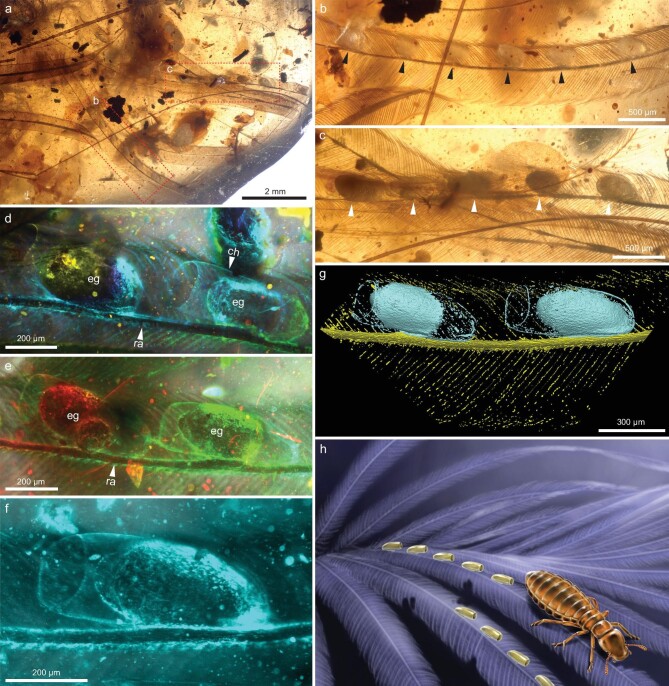
Fossilized nits of chewing lice, tightly affixed to feathers of an enantiornithine bird, entombed in mid-Cretaceous amber from northern Myanmar. (a) Isolated barbs and regularly arranged louse nits attached to two barbs of the same type. (b) Enlargement of six louse nits (indicated with black arrows). (c) Enlargement of five louse nits (indicated with white arrows). (d) and (e) Enlargement of louse nits. (f) Details of a nit, showing the placement, attachment and details of the chorion. (g) Enlargement of two louse nits, showing the attachments to ramus. (h) Ecological reconstruction of Cretaceous chewing lice of an enantiornithine bird. (a–c) Under normal reflected light. (d–f) Under confocal laser scanning microscopy. (g) Under X-ray micro-computed tomography. Abbreviations: ch, chorion; eg, egg; ra, ramus.

Lice constitute a distinct group of ectoparasites of mammals (including humans) and birds [13]. These wingless ectoparasitic insects spend their entire life cycle on the host's body. Currently, five major lineages of lice are recognized: Amblycera, Ischnocera, Trichodectera, Rhynchophthirina and Anoplura. Recent phylogenomic studies have provided compelling evidence that parasitic lice are nested within free-living bark lice, with the free-living Liposcelididae as the sister group of parasitic lice [13]. The discovery of a stem-group liposcelidid, *Cretoscelis*, dating back to the mid-Cretaceous in Myanmar, suggests that the divergence between parasitic lice and Liposcelididae occurred at least >99 million years ago [14]. Despite this, the initial hosts of lice remain elusive, with speculation including early mammals, early birds, potentially other feathered theropod dinosaurs, and even haired pterosaurs [7,14]. The presence of an Eocene crown-group bird louse provides direct evidence for a long-term coevolutionary history between lice and birds, hinting at the possibility of an ancestral host being an early feathered theropod dinosaur [8]. More recent advancements in phylogenomic studies [13] and ancestral-state reconstructions of parasitic lice lend support to an avian ancestral host, indicating that mammals acquired these parasites via host-switching from an ancient avian host. This hypothesis is supported by the earlier discovery of nits preserved in Eocene Baltic amber associated with mammalian hair [10], which establishes a minimum age for the transition from bird to mammal hosts.

Our identification of the earliest ectoparasitic louse nits on the feathers of enantiornithine birds holds significant implications for understanding the origin and early association between ectoparasites and their hosts. This discovery constitutes the most solid proof that lice expend their lifecycles on their hosts. Despite limited morphological information about the nits, the current fossil, discovered from the same deposit as the stem liposcelidids [14] and stem Amblycera [9], likely represents a stem group of parasitic lice, given that the earliest divergence between the extant lineages of lice is estimated to have occurred less than 99 Ma [13]. Indeed, little detail can be seen in the eggs that would indicate assignment to one of the five extant groups within Phthiraptera and we refrain from associating these nits with the stem-Amblycera adults recently reported from Kachin amber [9]. The chorion appears to lack any honeycomb or other ornamentation, which is otherwise common in many lice [15,16]. However, this ornamentation is often waxy and easily removed [17], and its apparent absence may be an artifact of preservation. There is no evidence of aeropyles or other structural ornamentation on the chorion, as is sometimes seen in some amblyceran eggs [18,19], but there is a hint of ornamentation in the form of seemingly regularly spaced pores or marks along the distal margin of the chorion.

The placement and attachment of the eggs on the feather are peculiar. Modern avian louse eggs are generally attached to the feather only at the basal end, sometimes forming a short stalk [20,21]. In contrast, the fossil eggs appear to be attached to the feather laterally by cement spread out over the basal three-fifths of the egg. This is uncommon in lice from extant birds, but occasionally reported [16]. More commonly, this form of extensive attachment is found in lice parasitizing mammals [22,23]. Additionally, the eggs are seemingly evenly distributed along the ramus, which is an unusual arrangement for avian louse eggs, although similar deposition patterns have been reported for a few avian lice [21].

Neither the placement, attachment, nor details of the chorion itself are typical of the eggs of modern avian lice, but each of the character states shown by these fossil eggs is known from at least some avian louse eggs. However, the oviposition and egg structure are unknown for most genera of extant lice, and appear to vary significantly within some groups [16]. Moreover, oviposition in extant avian lice is adapted to the existence of bird bills, which are the principal tool used for preening. In contrast, most enantiornithine birds had a toothed jaw [12], which may have imposed different selective pressures on oviposition in their lice. It is noteworthy that both these eggs and eggs of lice parasitizing toothed mammals have more extensive cemented attachment points. Modern avian lice can at times be quite specialized, living in microhabitats across a bird's body such as only on the wings, the head or amid body fluff. The current fossils correspond ecologically to those lice seemingly specialized for the shafts of downy feathers. Whether these same lice would have been found elsewhere on the body or were replaced by different species living elsewhere on the host remains an important topic of future exploration as it would indicate the antiquity of regional specialization and diversity among the earliest lice. More specimens of lice and louse eggs from various prehistoric hosts are needed to explore similarities and differences between extant and extinct groups of lice. Together with the recently discovered stem chewing lice in mid-Cretaceous amber, our discovery of the peculiar louse eggs of enantiornithine birds underscores the early origin of the association between ectoparasitic lice and their vertebrate hosts.

## Supplementary Material

nwae479_Supplemental_Files

## Data Availability

The original micro-Computed Tomography data are freely available at Science Data Bank: https://doi.org/10.57760/sciencedb.18931.
