# Supplementary material for: Cretaceous chewing-louse eggs on enantiornithine birds
Source: Natl Sci Rev. 2025 Jan 7;12(2):nwae479. doi: 10.1093/nsr/nwae479 (PMC11761765; doi:10.1093/nsr/nwae479)
Supplement: nwae479_Supplemental_Files [file nwae479_supplemental_files.zip › Supplemental_Data_Cai et al..docx]

**Supplementary Data**

**Cretaceous chewing louse eggs of enantiornithine birds**

Chenyang Cai^1^, Kevin P. Johnson^2^, Yanzhe Fu^1^, Daniel R. Gustafsson^3^, Dany Azar^1^, Yitong Su^1^, Qiang Xuan^1^, Michael S. Engel^4^, and Diying Huang^1,*^

^1^State Key Laboratory of Palaeobiology and Stratigraphy, Nanjing Institute of Geology and Palaeontology and Center for Excellence in Life and Paleoenvironment, Chinese Academy of Sciences, Nanjing 210008, China;

^2^Illinois Natural History Survey, Prairie Research Institute, University of Illinois, Champaign, IL, USA;

^3^Guangdong Key Laboratory of Animal Conservation and Resource Utilization, Guangdong Public Library of Wild Animal Conservation and Utilization, Institute of Zoology, Guangdong Academy of Sciences, Guangzhou 510260, China;

^4^Division of Invertebrate Zoology, American Museum of Natural History, New York, NY 10024-5192, USA.

∗Corresponding author.

Email: dyhuang@nigpas.ac.cn

Supplementary data include the following files:

Material and methods

Supplementary Figure 1

Supplementary Figure 2

**Materials and Methods**

The amber fossil reported in this study is part of the publicly accessible collections at the Nanjing Institute of Geology and Palaeontology, Chinese Academy of Sciences (NIGPAS, Nanjing, Jiangsu Province). Each amber piece in the collection of NIGPAS bears an informal field number, denoting the date and collector during its acquisition. The present specimen, with the field number HUANG-HP-B-3568, can be traced back to Prof. Diying Huang, who procured it in late 2016, predating local armed conflicts. The amber piece with the stem-group bird feather and louse nits was polished using sandpapers of gradually finer grits before finishing with diatomite powder. The photographs were taken using a Zeiss Discovery V20 stereo microscope. The confocal laser scanning microphotographs were taken using Zeiss LSM 710 with 10× objectives and using the 488 nm Argon laser excitation line. Helicon Focus v.7.0.2 were used to stack the images to increase the depth of field (Helicon Soft Ltd., https://www.heliconsoft.com/). Microtomographic data for two of the nits on the feather were obtained with a Zeiss Xradia 520 Versa 3D Xray microscope at the micro-CT laboratory of NIGPAS, and reconstructed in VGStudio MAX 3.0 (Volume Graphics Co., Ltd.). Scanning parameters were as follows: isotropic voxel size, 1.2 μm; power, 4 W; acceleration voltage, 50 kV; exposure time for each projection, 10 s; projections, 3201.


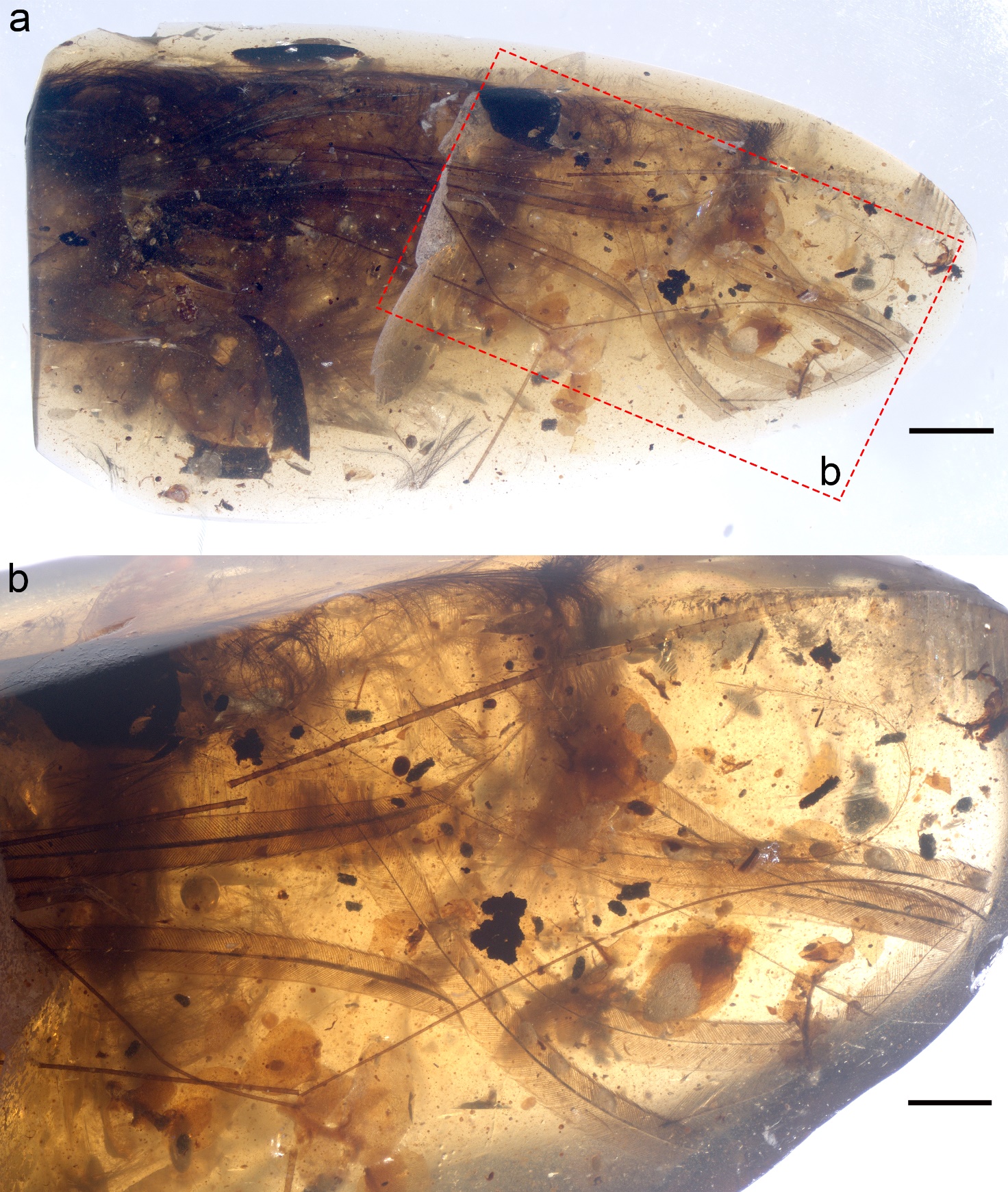


**Supplemental Figure 1.** Amber piece containing nits of chewing lice, from mid-Cretaceous of northern Myanmar. **a**, Amber piece in triangular shape. **b**, Enlargement of **a**, showing feathers of an enantiornithine bird and nits. Under normal reflected light. Scale bars: 2 mm in **a**, 1 mm in **b**.


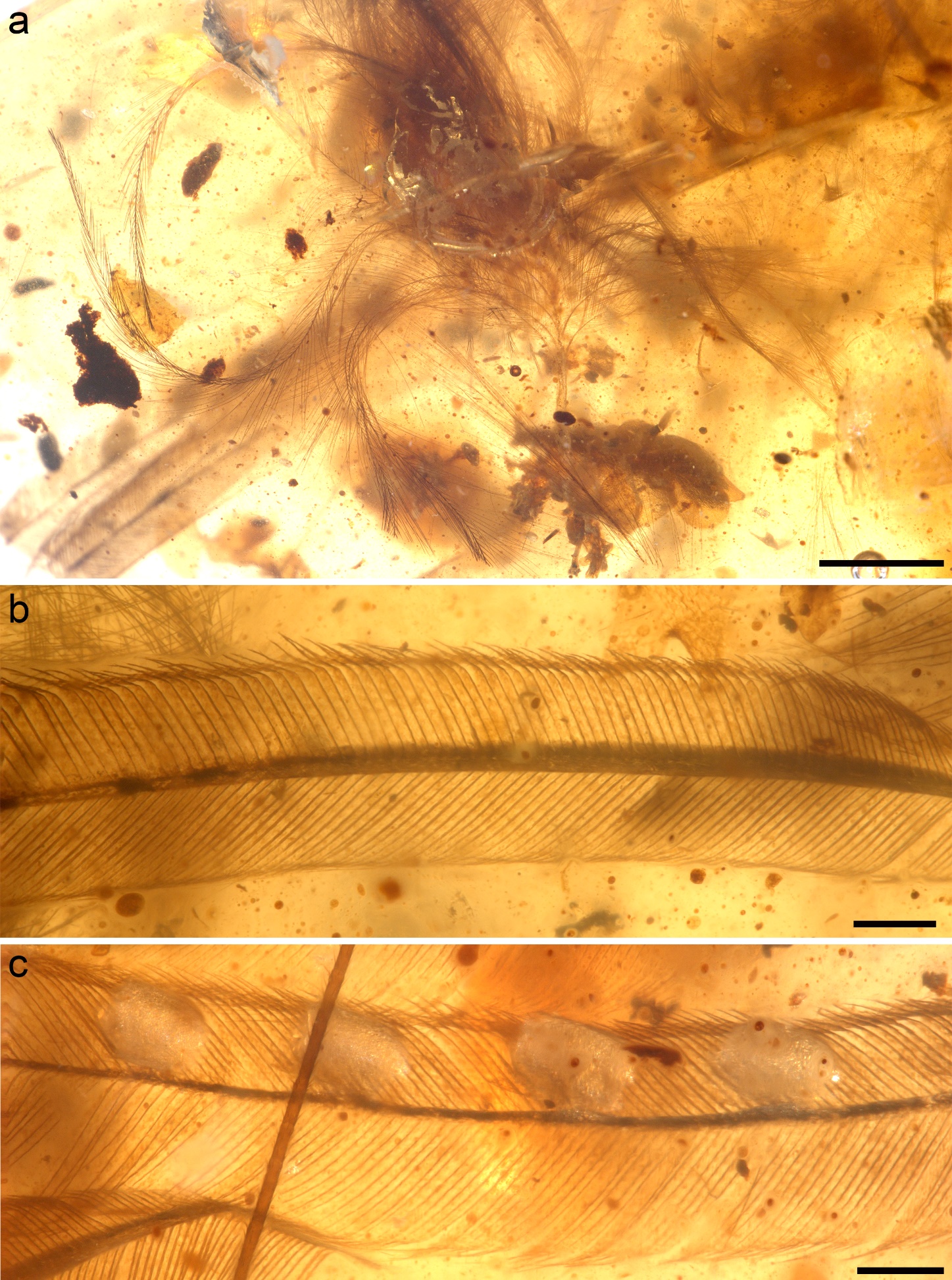


**Supplemental Figure 2.** Details of enantiornithine feather in mid-Cretaceous amber from northern Myanmar. **a**, Downy barb. **b**, Isolated barb. **c**, barb with four regularly spaced nits firmly attached to it. Under normal reflected light. Scale bars: 1 mm in **a**, 200 mm in others.
